# Acute long-acting anticoagulant rodenticide poisoning in pregnancy: a case report 

**DOI:** 10.3389/fphar.2024.1502596

**Published:** 2024-11-27

**Authors:** Yanxi Jia, Yongchi Zhan, Guiqiong Huang, Chunyan Deng, Haiyan Yu

**Affiliations:** ^1^ Department of Obstetrics and Gynecology, West China Second University Hospital, Sichuan University, Chengdu, China; ^2^ Key Laboratory of Birth Defects and Related Diseases of Women and Children (Sichuan University), Ministry of Education, Chengdu, China

**Keywords:** brodifacoum, long-acting anticoagulant rodenticides, vitamin K, pregnancy, coagulation dysfunction, case report

## Abstract

**Introduction:**

Brodifacoum is a highly potent superwarfarin rodenticide that leads to coagulopathy. Although the effect of warfarin during pregnancy is well understood, reports on superwarfarin poisoning, like brodifacoum, during pregnancy are scarce.

**Case Presentation:**

We report a case involving a woman with a singleton pregnancy who experienced sudden nasal hemorrhage accompanied by hematuria at 34 weeks and 3 days of gestation, with no apparent etiology. It was ultimately diagnosed as acquired coagulopathy resulting from brodifacoum poisoning. Fetal ultrasonography revealed significant intracranial hemorrhage, leading to intrauterine fetal demise and subsequent pregnancy termination. Following the correction of the patient’s coagulation profile through intravenous and oral administration of vitamin K1, tailored to the serum levels of brodifacoum, diligent monitoring confirmed that the patient was in stable condition.

**Conclusion:**

The primary hazard associated with the ingestion of brodifacoum is hemorrhage, with clinical presentations varying from asymptomatic cases to overt bleeding, which may present as hematuria, epistaxis, menometrorrhagia, and intracranial hemorrhage. Therefore, the diagnosis and management of this type of poisoning pose significant challenges. Prompt recognition and ongoing care for pregnant individuals affected by rodenticide toxicity are crucial for optimizing maternal health outcomes.

## Introduction

Superwarfarins, long-acting anticoagulant rodenticides (LAARs) derived from warfarin, are characterized by extended half-lives ranging from 24 to 120 days and enhanced potency in inducing coagulopathy compared to warfarin (Yaqoob et al., 2019). LAARs, like brodifacoum (BDF) and diphacinone-sodium, are common household hazards that can be ingested accidently or due to psychiatric disorders. In contrast to warfarin, LAARs demonstrate a high affinity for vitamin K epoxide reductase (VKOR), which is essential for the reactivation of vitamin K in the liver (King and Tran, 2015). The ingestion of LAARs results in the body’s inability to produce vitamin K-dependent coagulation factors, including factors II, VII, IX, and X, leading to an extension of prothrombin time (PT) and activated partial thromboplastin time (APTT).

The main risk of LAAR compound ingestion is bleeding, with symptoms ranging from asymptomatic to severe, including hematuria, epistaxis, menometrorrhagia, contusions, hemarthrosis, anemia, hemoptysis, and both retroperitoneal and intracranial hemorrhages. However, mucocutaneous bleeding sites are the most commonly reported bleeding sites, with hematuria being the most prevalent symptom (King and Tran, 2015). Studies shown that prothrombin time (PT) or international normalized ratio (INR) may be the most reliable screening test when conducted 48–72 h after exposure (Smolinske et al., 1989).

The diagnosis of LAAR poisoning depends on the careful selection and interpretation of laboratory tests. The levels of APTT, PT, and INR serve as initial indicators in the condition. If the results are disordered, the next step is to evaluate the activity levels of each individual factor, choosing between vitamin K-independent factors (typically FV) and vitamin K-dependent coagulation factors (FII, FVII, FIX, and FX). While these tests suggest vitamin K antagonism or deficiency, they do not confirm the presence of a specific agent. Only high-performance liquid chromatography (HPLC) can detect particular anticoagulant drugs. Blood or liver samples are mixed with a solvent and analyzed via HPLC (Vudathala et al., 2010).

While the impact of warfarin in pregnancy is well-documented, instances of superwarfarin toxicity, particularly from BDF, during gestation are limited. Warfarin, a common anticoagulant, is contraindicated in pregnancy due to its placental transfer and associated embryopathy risks, such as midface hypoplasia and limb deformities. Later use can lead to fetal intracranial hemorrhage and schizencephaly. In contrast, unfractionated heparin and low-molecular-weight heparin do not cross the placenta, making them the preferred anticoagulants during pregnancy (Bulletins, 2018; Kolettis and Craigo, 2018).

This case report details a woman with a singleton pregnancy who presented with sudden onset nasal hemorrhage and hematuria at 34 weeks and 3 days of gestation, which was linked to acquired coagulopathy resulting from BDF toxicity. Fetal ultrasound examination revealed significant intracranial hemorrhage, ultimately resulting in intrauterine fetal demise and the necessity for pregnancy termination. After the patient’s coagulation parameters were normalized through intravenous and oral administration of vitamin K1, guided by serum BDF concentrations, diligent monitoring confirmed the patient’s stable condition.

Additionally, a comprehensive literature review was conducted utilizing keywords such as BDF and pregnancy in both English and Chinese contexts (as shown in Table 1) (Zurawski and kelly, 1997; Mehlhaff et al., 2013; Yan et al., 2013; Ma et al., 2017). Currently, there have been a total of four reported cases of long-acting anticoagulant rodenticide poisoning during pregnancy that were similar to our case. Most of the affected pregnant women presented with hematuria, epistaxis, or mucocutaneous bleeding, and their fetuses succumbed to intracranial hemorrhage. Each of the pregnant women received the treatment of vitamin K, and subsequent follow-ups indicated a restoration of normal coagulation function. This study received approval from the Ethics Committee of West China Second University Hospital.

**TABLE 1:** Summary of the clinical features of cases with rodenticide poisoning during pregnancy.

Study id	Age	GA	Clinical presentation	Fetus’ condition	Blood level of rodenticide	Treatments		Maternal outcomes
						Fetus	Pregnant woman	
Zurawski and kelly (1997)	19	22	blood in the oropharynx, vaginal bleeding, and hematuria	With normal fetal heart rate and without evidence of fetal hemorrhage	BDF: 220 ng/mL	Careful antenatal care	intravenous and oral vitamin K	At term, a viable 3120 g male infant was delivered Vaginally. Normal coagulation studies of both the mom and baby
Mehlhaff et al. (2013)	19	32	Spontaneous mucosal bleeding	acidosis	NS	ECS	intravenous and oral vitamin K	The neonate died at the 4th day after ECS because of intracranial hemorrhage, and the pregnant woman in good condition after the treatment
Yan et al. (2013)	21	37	gross hematuria	intracranial hemorrhage	BDF: 1310 ng/mL	NS	intravenous prothrombin complex, FFP, intramuscular vitamin K	The neonate was delivered stillborn and the pregnant woman in good condition after the treatment during the 6 month follow-up
Ma et al. (2017)	25	38	bleeding of oral mucosa	intracranial hemorrhage	BRD: 126 ng/mL	ECS	vitamin K intravenously	The pregnant woman in good condition after the treatment
Our case	24	34^+3^	nasal bleeding and hematuria	intracranial hemorrhage	BDF: 337.5 ng/mL	labor induction	prothrombin complex, FFP, intravenous and oral vitamin K	The pregnant woman in good condition after the treatment

* GA: gestational age; BDF: brodifacoum; BRD: bromadiolone; ECS: emergency cesarean section; FFP: Fresh-frozen plasma; NS: not specified.

## Case presentation

A 24 year-old female patient (gravida 1, para 0) with a singleton pregnancy conceived naturally was recently admitted to our hospital after experiencing sudden nasal hemorrhage and hematuria at 34 weeks and 3 days of gestation, with no identifiable causes. Laboratory results indicated a PT of 73.55 s, APTT of 42.61 s, and an INR of 6.13. Her social and family histories were unremarkable; however, her medical history noted an episode of unexpected nasal bleeding at 33 weeks and 4 days of gestation. The bleeding was resolved following the nasal application of bovine basic fibroblast growth factor gel.

The recurrent hemorrhage was significant, exhibiting a greater volume than previous one, and nasal packing proved ineffective at this time. However, there were no discernible bleeding sites or petechiae in the subcutaneous tissue throughout the body, nor were there any indications of abdominal pain or vaginal bleeding. Laboratory analyses indicated reductions in the activities of coagulation factors II (3.5%), VII (8.7%), IX (1.4%), and X (1.2%), alongside a prolonged APTT of 95.7 s, while factors V, VIII, and XI were found to be within the normal range. Imaging studies, including urinary ultrasound, echocardiogram, chest ultrasound, and liver ultrasound, showed no apparent abnormalities in the patient. Additionally, fetal heart monitoring demonstrated a stable baseline fetal heart rate with minimal variability and deceleration, while fetal ultrasonography revealed evidence of intracranial hemorrhage. The patient’s unexplained bleeding posed a diagnostic dilemma, necessitating the implementation of blood component therapy, including fresh-frozen plasma (FFP), prothrombin complex concentrate (PCC), hemostatic agents, and vitamin K. Unluckily, the patient and her family opted to forgo further intervention for the fetus, citing the mother’s critical health status and financial constraints as primary factors in their decision.

Due to the patient’s critical condition, a series of coagulation tests were conducted by a multidisciplinary team (MDT) including obstetricians, hematologists, and pharmacists. The examinations revealed normal levels of fibrinogen, D-dimer, fibrin degradation products (FDP), von Willebrand factor, lupus anticoagulant, and antithrombin. Serological tests for antinuclear antibodies, anti-dsDNA antibodies, and anticardiolipin antibodies were negative, as was the antiglobulin (Coombs') test. The introduction of normal plasma in a 1:1 ratio corrected the coagulation abnormalities (APTT test), suggesting a coagulation factor deficiency. We hypothesized a potentially life-threatening coagulation disorder linked to deficiencies in vitamin K–dependent proteins. The differential diagnosis at this stage includes: (1) vitamin K deficiency; (2) hereditary deficiency of vitamin K–dependent carboxylase or vitamin K epoxide reductase; or (3) accidental or covert ingestion of a vitamin K antagonist, such as warfarin, phenprocoumon, or a potent rodenticide (“superwarfarin”).

**FIGURE 1:**
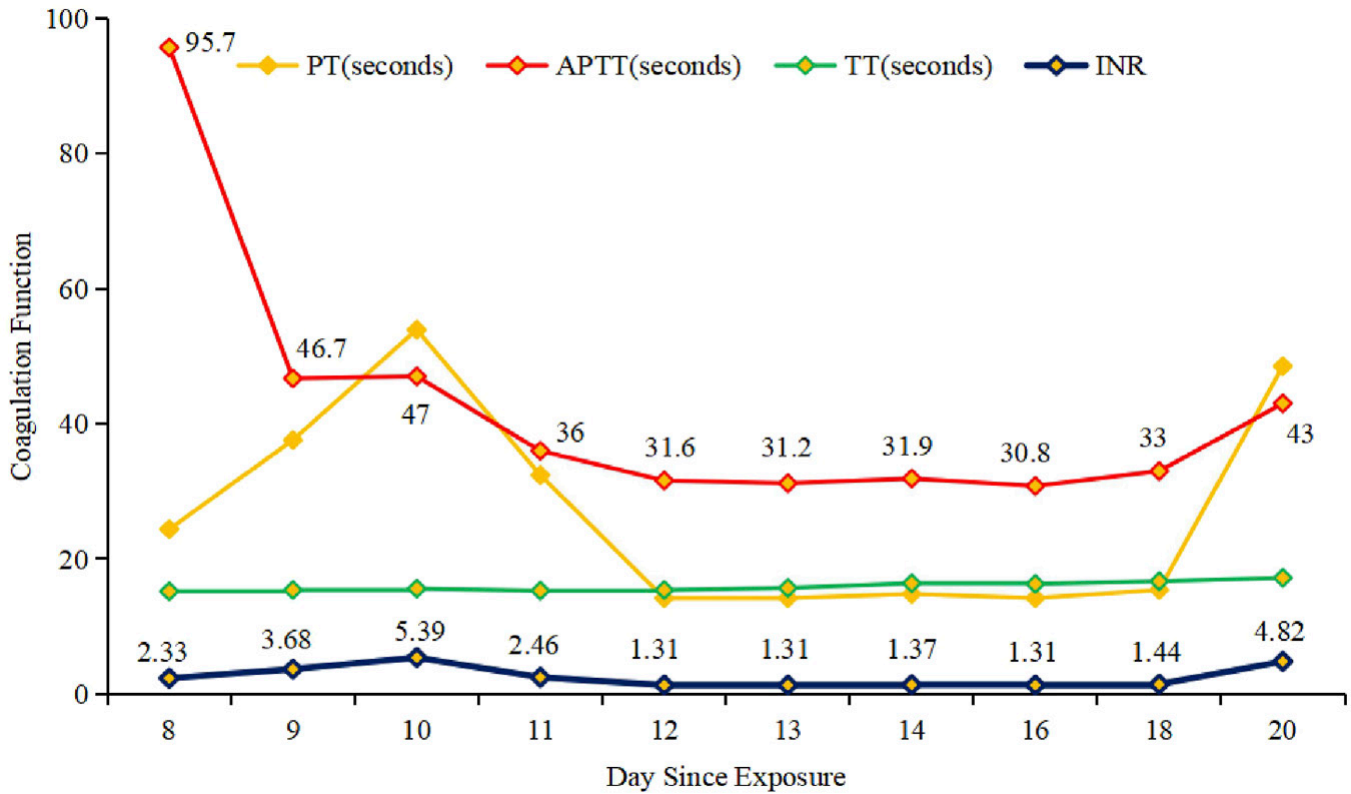
Coagulation function and usage of vitamin K1 over time.

Ultimately, the patient’s blood tested positive for BDF at 337.5 ng/mL. A detailed history revealed unintentional rodenticide exposure 2 days prior to symptom onset. If BDF poisoning is suspected, prompt coagulopathy correction with FFP and vitamin K is crucial for effective maternal resuscitation (Gunja et al., 2011). Thus, FFP and vitamin K1 were administered to reverse the coagulopathy status (Figure 1). However, it is important to highlight that currently, there are no established protocols regarding the administration and dosage of vitamin K1 in cases of poisoning. In this context, the decision to modify treatment approaches and the specific dosing schedule were informed by the clinical experience of hematologist, as well as the blood levels of BDF and the patient’s coagulation status.

**FIGURE 2:**
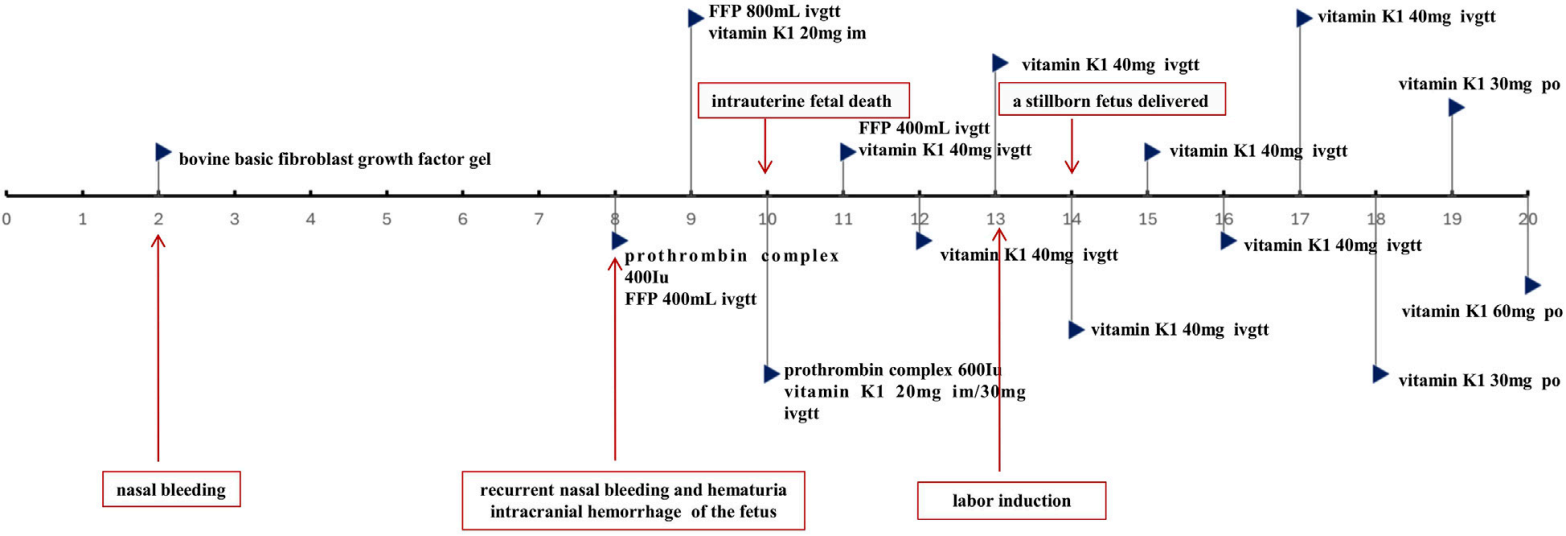
Timeline of the treatments and key events. *FFP:fresh-frozen plasma; im: intramuscular injection; ivgtt: intravenous drip; po: per os.

**FIGURE 3:**
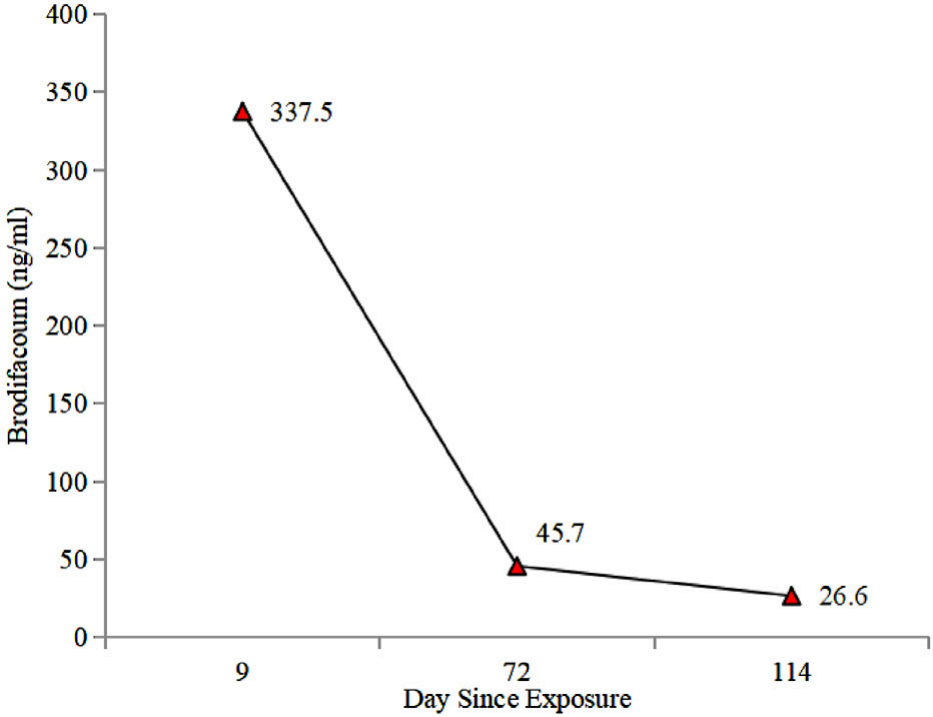
Brodifacoum concentration in patient’s peripheral blood over time.

At 34 weeks and 5 days of gestation (the 10th day of BDF exposure), the fetal ultrasound indicated significant intracranial hemorrhage, ultimately leading to intrauterine fetal death. Following the correction of the active coagulation function, the patient exhibited no apparent bleeding tendency by the 13th day of BDF exposure. After consultations with obstetricians and hematologists, labor induction was initiated. On the 14th day of BDF exposure, the patient delivered a stillborn fetus. We continuously monitored the patient’s coagulation function in relation to the duration of BDF exposure (Figure 1). Additionally, we constructed a timeline detailing the patient’s treatments, the concentration of BDF in the maternal bloodstream, and her health status during the follow-up (Figures 2, 3).

## Discussion

BDF, a long-acting vitamin K antagonist rodenticide, is associated with intentional ingestions and contamination in synthetic cannabinoids, leading to prolonged PT and APTT (Moritz et al., 2018). The evidence of extended PT, APTT, and INR, along with significantly reduced vitamin K-dependent coagulation factors, complicates differential diagnosis between hemophilia and acquired hemophilia (AH). Hemophilia A and B are congenital disorders due to deficiencies in clotting factors VIII and IX, respectively, with female cases being rare due to the recessive X-linked inheritance. Women are often heterozygous carriers, exhibiting reduced clotting factor levels and mild symptoms. A family history of spontaneous bleeding aids in diagnosing hemophilia (Berntorp et al., 2021). AH, a rare condition caused by autoantibodies against endogenous factor VIII, is typically identified *postpartum*. IgG antibodies can cross the placenta, heightening the neonate’s hemorrhage risk. Corticosteroids are the first-line treatment for pregnancy-related AH (Berntorp et al., 2021). However, the patient in our case denied any familial or genetic history.

Pregnant women with spontaneous bleeding should be assessed for coagulopathy through history and laboratory tests, including complete blood count, PT, APTT, fibrinogen, and metabolic panel. Differential diagnoses include obstetric coagulopathies like disseminated intravascular coagulation (DIC) with abruption and fetal demise, fulminant liver failure, hereditary deficiencies, and drug effects. Abnormalities in both intrinsic and extrinsic pathways indicate a common pathway deficiency or inhibition of vitamin K-dependent factors. Therefore, in cases of severe painless hematuria, after excluding other conditions, impaired coagulation possibly due to poisoning should be considered.

In our case, the woman’s bleeding symptoms ranged from nasal bleeding to hematuria, resembling pregnancy complications due to DIC. However, her hematuria persisted for several days, which DIC could not explain. Additionally, routine blood tests, liver function tests, fibrinogen levels, fibrinogen degradation product (FDP), and D-dimer were normal, consistent with previous LAAR poisoning cases (Yan et al., 2013). Clinically significant LAAR poisoning is often challenging to identify due to: 1) its prevalence in adults who intentionally or covertly expose themselves; 2) delayed symptom onset post-exposure; and 3) variability in symptoms among individuals.

Previous studies indicate that the primary manifestation of BDF poisoning is spontaneous bleeding, including nosebleeds, hematuria, and mucosal bleeding (Mehlhaff et al., 2013). A review of 174 LAAR exposure cases identified hematuria, gingival bleeding, epistaxis, and gastrointestinal bleeding as the top four hemorrhagic features. Notably, intracranial hemorrhage was the leading cause of death, with BDF being the most common agent in LAAR exposures (King and Tran, 2015).

According to earlier reports, maternal-fetal outcomes from BDF poisoning vary from healthy fetuses to intracranial hemorrhaging or abortion, depending on pregnancy stage (Lipton and Klass, 1984; Zurawski and Kelly, 1997; Tosounidou and Gordon, 2019; Rubinstein et al., 2018). There is no specific information on whether BDF crosses the placenta or is present in breast milk. We have summarized the clinical manifestations and maternal outcomes of reported cases, including our case of LAARs poisoning during pregnancy (Table 1). The first case (Zurawski and Kelly, 1997) in 1997 involved a 19 year-old woman at 22 weeks gestation, experienced oral mucosa bleeding, vaginal bleeding, and hematuria, yet delivered vaginally at term with normal coagulation studies for both mother and baby. The absence of fetal hemorrhage led investigators to hypothesize that BDF might not cross the placenta. However, subsequent cases showed all fetuses had intracranial hemorrhage and died, contradicting the initial hypothesis. Possible explanations for the lack of fetal hemorrhage in the first case include: 1) BDF crosses the placenta but its effects are mitigated by maternal vitamin K treatment; 2) BDF may have teratogenic effects that are influenced by individual fetal factors.

LAARs tend to accumulate in the liver, exhibiting prolonged half-lives due to increased lipid solubility and slow metabolism in specific areas. Accordingly, vitamin K1 maintenance treatment is often necessary post-LAAR poisoning, as premature cessation can result in abnormal coagulation and bleeding, even with normal coagulation tests. In our case, we reduced the vitamin K1 dosage on the 18th day of exposure, leading to evident coagulation abnormalities in the patient.

Previous clinical experiences indicate that BDF poisoning is primarily managed with vitamin K1, though treatment duration varies based on blood concentration and coagulation status, complicating management. Vitamin K1 acts as a specific antidote for LAAR poisoning, rapidly addressing intrinsic and extrinsic coagulation pathway abnormalities, with normalization of coagulation parameters (PT, APTT, INR) typically occurring within 2, 3 days post-treatment (Tsutaoka et al., 2003).

However, vitamin K1 may also induce thrombotic complications, such as a popliteal deep vein thrombosis (Franco et al., 2013) and intracranial venous thrombosis (Papin et al., 2007), likely due to early depletion of anticoagulant proteins C and S (Lipton and Klass, 1984). Other rare adverse events include angioedema with airway compromise, diffuse alveolar hemorrhage, hemarthrosis, miscarriage, and bowel obstruction due to bowel wall hematoma (Barnett et al., 1992).

In non-pregnant cases, while vitamin K1 can reverse LAAR-induced coagulopathy, this process requires at least 12 h (Haesloop et al., 2016). Patients experiencing active bleeding may benefit from PCC (II, VII, IX, and X) infusion; a case study reported normalization of INR within 30 min following 2000 U of PCC in BDF toxicity patients (Doyle et al., 2018). Another study noted rapid and sustained coagulopathy reversal in a LAAR poisoning patient after receiving 45 IU/kg PCC.

The timing for discontinuing vitamin K1 therapy in LAAR poisoning remains contentious, Experts recommend using HPLC to track the serum levels to determine the course of treatment. Furthermore, the elimination kinetic can be plotted and a treatment course determined using the BDF concentration in the peripheral blood. However, in clinical practice, treatment has often been discontinued arbitrarily, with subsequent monitoring of the coagulation profile. A retrospective study of 21 LAAR poisoning patients analyzed serum rodenticide levels before and after vitamin K1 treatment, revealing that those with concentrations >10 ng/mL were at higher risk for re-bleeding (Rubinstein et al., 2018). For BDF poisoning, it is suggested that vitamin K1 can be safely discontinued at serum concentrations <10 ng/mL (Bruno et al., 2000). Whereas, low circulating BDF levels (<10 ng/mL) can still be detected in cases, the harmful effects of these concentrations on fetal development remain unclear. Future multicenter, randomized controlled studies with larger sample sizes are needed to further validate our findings regarding treatment duration, dosing, and withdrawal timing.

A report revealed the development of a straightforward fluorescence micro-plate immunoassay utilizing an *in situ* fluorogenic reaction for detecting bromadiolone (BRD) and BDF in food and biological matrices, demonstrating significant potential for food safety monitoring and BRD/BRF poisoning diagnostics (Li et al., 2024).

Blood perfusion theoretically could eliminate LAAR due to its large molecular weight and high fat solubility; however, further research is needed to assess its applications and therapeutic benefits. As a cytochrome P450 inducer, phenobarbital enhances liver microsome metabolism and the detoxification capacity of liver enzymes, yet its effect on BDF metabolism or clearance remains unclear (Rubinstein et al., 2019).

Acute hemorrhagic symptoms typically necessitate intravenous vitamin K1 doses exceeding 50–100 mg, while chronic maintenance often involves 100 mg of oral vitamin K1 daily to manage coagulopathy, with treatment durations averaging 168 days. Adjunctive hemostatic therapies, including recombinant factor VIIa and PCC, have been documented, and phenobarbital has been employed to accelerate LAAR metabolism (King and Tran, 2015).

It is clear that appropriate blood products (FFP/red blood cells) and intravenous or oral vitamin K1 are essential treatments for BDF-induced coagulopathy and bleeding. Furthermore, the administration and dosage of vitamin K1 must be tailored to the drug concentration and coagulation status. Post-discharge, we meticulously monitored BDF levels in peripheral blood (Figure 2) and adjusted the treatment plan according to the hematologist’s guidance.

In summary, two primary challenges in managing long-acting anticoagulant rodenticides are diagnosis difficulty and coagulopathy correction, particularly in pregnant women. Treatment for these cases may involve: differential diagnosis, bleeding control (coagulation correction), vitamin K antagonist therapy, long-term follow-up, and psychosocial evaluation. Severe bleeding in previously healthy patients or laboratory signs indicating high bleeding risk poses diagnostic challenges. Rapid assessment of prolonged PT and PTT, along with specific vitamin K-dependent coagulation deficiencies, significantly narrows the differential diagnosis. Given the life-threatening nature of this condition and its resolution with proper treatment, swift and precise diagnosis is essential.

It is important to recognize that our study is constrained by the limited availability of BDF concentrations in the placenta, amniotic fluid, and the fetus, which could not be collected following fetal death attributed to intracranial hemorrhage. Thus, it remains uncertain whether the fetal death resulting from intracranial hemorrhage is linked to acquired coagulation dysfunction induced by BDF. This further substantiates the notion that BDF may influence fetal coagulation function via the placenta.

## Conclusion

Bleeding is the major risk following ingestion of BDF, and the clinical manifestations range from being asymptomatic to active bleeding manifested as hematuria, epistaxis, menometrorrhagia, and intracranial hemorrhage. Therefore, diagnosis and treatment of this intoxication remain a challenge. Early identification and long-term management of pregnant women with rodenticide poisoning play a vital role in the maternal outcomes.

## Data Availability

The original contributions presented in the study are included in the article/supplementary material, further inquiries can be directed to the corresponding author.
